# Eosinophilic fasciitis: A case report and literature review

**DOI:** 10.1097/MD.0000000000045449

**Published:** 2025-10-24

**Authors:** Yongping Dang, Limin Niu, Dandan Wang, Yuanhao Li, Keyan Qian, Haijun Ma, Minghao Guo, Jin Li

**Affiliations:** a Department of Nephrology, The First Affiliated Hospital of Xinxiang Medical University, Xinxiang, China.

**Keywords:** eosinophilia, eosinophilic fasciitis, groove sign, sclerosis

## Abstract

**Rationale:**

Eosinophilic fasciitis (EF) is a rare fibrosing disorder with an undetermined etiology and an incompletely elucidated pathogenesis. Although eosinophilia is frequently observed during the acute phase and serves as a diagnostically suggestive feature, its absence does not exclude active disease. Notably, the incidence of eosinophilia shows a progressive decline in chronic cases, correlating with the disease duration and fibrotic progression.

**Patient concerns:**

A 42-year-old male manual laborer presented with a 12-month history of progressive symmetric induration and hyperpigmentation affecting all 4 limbs.

**Diagnoses:**

Blood tests revealed eosinophilia (1.52 × 10⁹/L), and magnetic resonance imaging demonstrated characteristic hyperintensity of the fascial layers on T2-weighted imaging.

**Interventions:**

The patient was initially treated with low-dose corticosteroids combined with methotrexate.

**Outcomes:**

Laboratory monitoring confirmed complete normalization of the previously elevated peripheral eosinophil count, indicating a favorable treatment response.

**Lessons:**

EF is a rare connective tissue disorder that frequently poses diagnostic challenges, often leading to missed or incorrect diagnoses in clinical practice. However, contemporary diagnostic approaches enable the accurate identification of suspected EF cases through comprehensive evaluation of characteristic clinical manifestations, laboratory findings, and imaging features, even in the absence of confirmatory biopsy.

## 1. Introduction

Eosinophilic fasciitis (EF) is an uncommon fibrotic disorder of the connective tissue, affecting fewer than 0.01% of the population.^[1]^ Current diagnostic standards support a multimodal strategy incorporating clinical, histopathological, and imaging criteria. Nevertheless, confirmatory biopsy is not attainable in 20% to 30% of patients owing to practical constraints.^[2]^ We present a case of EF exhibiting classic features, in which the diagnosis was reached despite the absence of histopathological confirmation. A standardized treatment approach was implemented based on a comprehensive assessment of clinical presentation, laboratory results, and imaging features. Following conservative management, the patient’s condition improved significantly.

## 2. Case report

A 42-year-old male manual laborer presented with a 12-month history of progressive symmetrical induration and hyperpigmentation involving all 4 extremities. The condition initially manifested in the lower limbs (characterized by painful swelling and cutaneous thickening of the bilateral calves), with subsequent proximal progression to the upper extremities (forearms) over a 6-month period. The patient exhibited complete absence of inflammatory arthralgia or Raynaud phenomenon. No family history of heritable connective tissue disorders was reported. The patient denied tobacco use and significant alcohol consumption. Due to the progressive nature of his symptoms, this manual laborer experienced a complete occupational disability.

**Figure 1.**
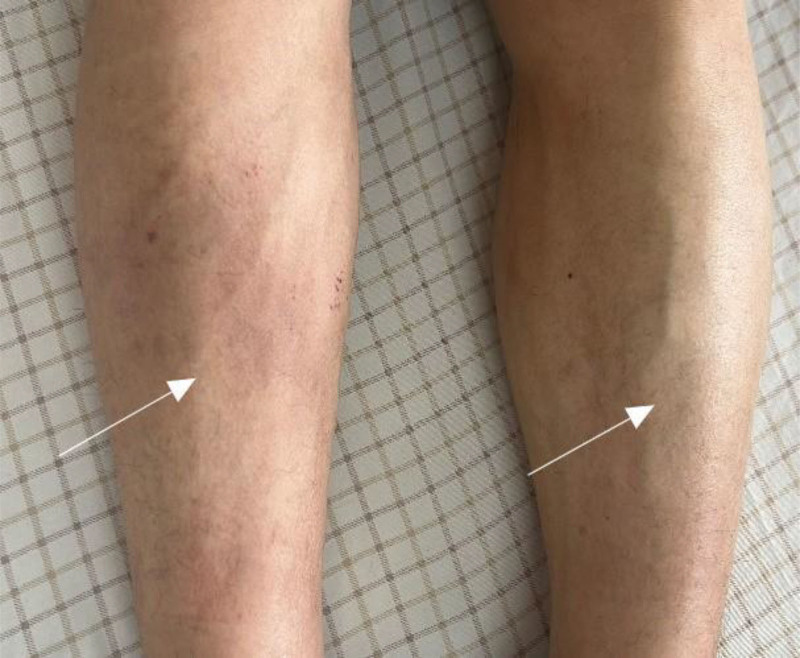
Bilateral lower extremity edema and sclerosis were observed.

**Figure 2.**
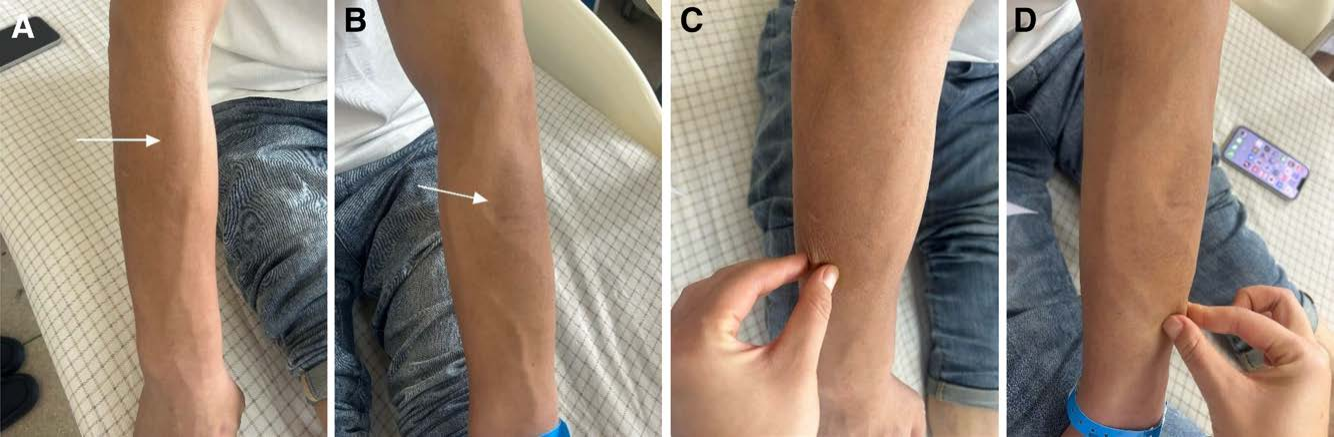
(A and B) Visible linear depressions along superficial venous tracts; (C and D) limitation in skin mobility over the bilateral forearms.

Physical examination revealed pathognomonic findings consistent with EF, including symmetrical induration of the distal forearms and lower extremities along with the diagnostic groove sign (Figs. 1 and 2A–D).

Laboratory investigations revealed eosinophil percentage (17.60%, reference range 0.4–8%), blood eosinophilia (1.52 × 10^9^/L, reference range < 0.5 × 10^9^/L), erythrocyte sedimentation rate (ESR) (19 mm/h, reference range < 15 mm/h), C-reactive protein (CRP) (15.20 mg/L, reference range < 10 mg/L). Autoantibodies to systemic sclerosis (SSc), antinuclear antibody, immunoglobulin A, E, and M were negative, with no complement consumption. Electromyography revealed no evidence of myositis or polyneuropathy.

**Figure 3.**
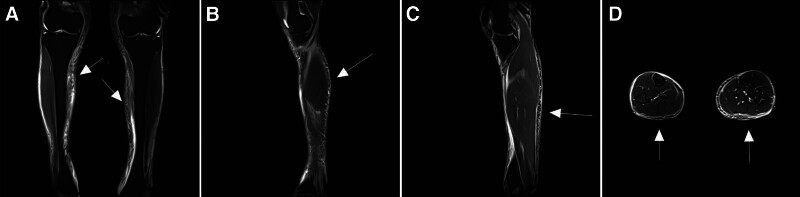
Lower limb MRI. (A) Coronal T2WI, both calves. (B) Sagittal T2WI, right calf. (C) Sagittal T2WI, left calf. (D) Axial T2WI, both calves. MRI = magnetic resonance imaging, T2WI = T2-weighted imaging.

Magnetic resonance imaging (MRI) demonstrated a characteristic hyperintensity of the fascial layers on T2-weighted imaging, indicative of active inflammatory involvement (Fig. 3A–D).

The patient was initially treated with low-dose corticosteroids (25 mg oral prednisolone daily) in combination with methotrexate (10 mg weekly). After 2 months, marked regression of dermal induration was observed in the upper extremities, accompanied by modest improvement in the lower limbs. Concurrently, the previously noted peripheral eosinophilia had completely normalized. Prednisolone tapering was initiated after, 1 year of treatment. The patient remained under active surveillance.

## 3. Discussion

EF was initially reported by Shulman in 1974.^[3]^ It is an uncommon connective tissue disorder presenting with symmetrical indurated edema, erythematous cutaneous lesions, and subcutaneous induration, most frequently observed in the lower legs and forearms. In the present case, the patient demonstrated typical manifestations of EF, including bilateral involvement in these characteristic anatomical sites. Diagnostic signs such as the “groove sign” or a peau d’orange appearance are seen in approximately one-third to half of all cases. In more advanced stages, complications may include joint contractures or neurological involvement.

The etiology of EF is considered multifactorial, with reported triggers encompassing repetitive strenuous exertion, autoimmune abnormalities, radiation, genetic susceptibility, hemodialysis for end-stage renal disease, malignancy, and certain pharmacological agents.^[4]^ Our patient worked as a technician performing repetitive manual tasks over an extended period and had no other clear predisposing factors. Research indicates that intense physical activity can induce interleukin (IL)-33 release from vascular endothelium, leading to activation of type 2 innate lymphoid cells and potentially facilitating fasciitis.^[5]^ Based on this mechanistic insight and the patient’s occupational history, we suggest that prolonged mechanical stress may have been a key contributing factor in this instance.

The underlying causes of EF are not fully understood, though growing evidence points to autoimmune processes as major contributors to disease development. Histopathological examinations of EF patients show substantial increases in fibronectin and collagen type 1 alpha 1 chain within dermal fibroblasts. Fibrosis in EF is thought to arise mainly from overproduction of tissue inhibitor of tissue inhibitor of metalloproteinases 1, which physiologically inhibits matrix metalloproteinase-1.^[6]^ Eosinophilia is a defining feature of EF, supported by raised serum levels of eosinophil cationic protein and IL-5, as well as heightened chemotaxis and migration of eosinophils.^[7,8]^ Elevated plasma histamine also suggests involvement of mast cells.^[9]^ Immunological analyses reveal strong activation of peripheral blood mononuclear cells in individuals with EF, marked by increased release of IL-2, interferon-γ, and leukemia inhibitory factor, continued high expression of CD40 ligand, and notable rises in superoxide dismutase activity. These patterns align with abnormal activation of T helper cells.^[10]^ On a molecular level, research shows increased transforming growth factor-β1 mRNA in fibroblasts isolated from fascia, as well as elevated CTGF gene expression in diseased fascial samples.^[11]^ Collectively, these results emphasize the importance of profibrotic cytokines in EF pathophysiology.

Laboratory findings in EF often reveal transient peripheral eosinophilia, though its degree does not reliably reflect disease activity 60% to 80% of patients show elevations in acute-phase reactants (including ESR and CRP) as well as hypergammaglobulinemia.^[12]^ Notably, blood eosinophilia can be brief, and tissue eosinophil clearance may occur even before peripheral counts normalize. Therefore, when clinical suspicion for EF is high, additional investigations such as biopsy or MRI should be considered despite normal eosinophil levels.^[13]^ Among patients with active disease, around 70% present with elevated serum aldolase. The serum level of procollagen type III aminoterminal peptide, which reflects collagen synthesis, correlates with disease activity and may assist in monitoring treatment response.^[14]^ Although autoantibodies are not hallmark features of EF, low titers of anticentromere, anti-topoisomerase I, or anti-RNA polymerase III antibodies are occasionally detected in 15% to 20% of cases. Anti-neutrophil cytoplasmic antibody testing is useful to exclude eosinophilic granulomatosis with polyangiitis. About 10% of patients test positive for antinuclear antibody or rheumatoid factor.^[15]^ In situations where histopathology is unavailable or nondiagnostic, MRI of symptomatic areas provides important diagnostic clues. Typical imaging features comprise increased T2 signal in subcutaneous tissue and deep fascia, together with strong fascial enhancement on post-contrast fat-suppressed T1-weighted imaging, suggesting active inflammation of the fascia.

The following disorders were carefully evaluated and excluded during the differential diagnosis process using clinical, serological, and electrophysiological assessments: first, SSc involves cutaneous fibrosis affecting subcutaneous and fascial tissues, and is commonly accompanied by anti-Scl-70 antibodies and Raynaud phenomenon (reported in approximately 95% of patients triggered by cold or emotional stress).^[16]^ This patient tested negative for anti-Scl-70 antibodies and had no history of Raynaud, effectively ruling out SSc. Second, dermatomyositis is typified by classic skin findings including heliotrope rash and Gottron papules, as well as progressive proximal muscle weakness. Typical laboratory features consist of anti-Jo-1 antibodies, elevated creatine kinase, and electromyographic abnormalities, with some cases associated with underlying malignancy.^[17]^ No typical skin lesions were observed here, serial creatine kinase measurements remained normal, and electromyographic showed no signs of myopathic or neurogenic damage, making dermatomyositis an unlikely diagnosis. Third, eosinophilia–myalgia syndrome is frequently related to contaminated L-tryptophan ingestion and generally presents with pronounced eosinophilia, intense myalgia, and multisystem manifestations such as cutaneous, cardiovascular, or neurological involvement.^[18]^ There was no history of L-tryptophan use in this case, and neither clinical nor laboratory findings indicated multi-organ involvement, supporting the exclusion of eosinophilia–myalgia syndrome.

The development of validated diagnostic criteria for EF remains an important goal in clinical research. International guidelines currently advocate for a multidimensional diagnostic strategy that combines clinical presentation, laboratory studies, imaging results, and histopathological data when available. MRI has become the preferred imaging method in EF evaluation, offering practical benefits including targeted biopsy guidance, objective measurement of disease activity, and assessment of therapeutic response.^[19,20]^ In 2014, Pinal-Fernandez et al introduced a set of diagnostic criteria derived from a multicenter investigation^[21]^ (Table 1). For this patient, while tissue biopsy was not performed, the diagnosis was substantiated by fulfilling 1 major criterion (distinctive skin changes) and 3 minor criteria (peripheral eosinophilia, hypergammaglobulinemia, and characteristic MRI patterns), in addition to systematic exclusion of other conditions and a pronounced clinical improvement following treatment with glucocorticoids and immunosuppressive agents. This strategy embodies the modern notion of “diagnostic therapy” and provides a practical example of a noninvasive diagnostic pathway for EF. Thus, by combining 5 key elements (clinical features, imaging observations, laboratory indicators, treatment response, and differential diagnosis exclusion) clinicians can confidently diagnose EF without mandatory histological confirmation, promoting earlier treatment initiation, and possibly enhancing clinical outcomes.

**Table 1:** Diagnostic criteria of eosinophilic faciitis.

*Major criterion*: (1) Cutaneous and subcutaneous involvement: symmetric or asymmetric, diffuse (affecting limbs/trunk/abdomen) or localized (limbs only) swelling, induration, and thickening of the skin and subcutaneous tissues. (2) Histopathological features: fascial thickening with lymphocytic and macrophage infiltration (eosinophilic infiltration was not required).
*Minor criteria*: (1) Peripheral blood eosinophilia. (2) Hypergammaglobulinemia (serum level > 1.5 g/L). (3) Muscle weakness with or without elevated aldolase levels. (4) Characteristic skin changes (“groove sign” or “peau d’orange” appearance). (5) High signal intensity in the fascial layers on T2WI.
After exclusion of systemic sclerosis, the diagnosis can be established by either (1) meeting both major criteria or (2) fulfilling 1 major criterion plus at least 2 minor criteria

T2WI = T2-weighted imaging.

There is currently no universally accepted treatment protocol for EF. Management typically involves glucocorticoids, frequently combined with immunosuppressive drugs. The initial therapy, as per established guidelines, is prednisone at a dosage of 1 mg/kg/d.^[6]^ Once laboratory values (such as eosinophil count, ESR, and CRP) return to normal, a gradual reduction in corticosteroid dosing is recommended. While these markers often respond quickly to treatment, full resolution of cutaneous symptoms usually requires continued therapy over weeks or months. In patients who do not respond adequately to glucocorticoids or cannot tolerate them, the addition of immunosuppressive agents is advised. Methotrexate is generally preferred as the first-choice immunosuppressant, given at 15 to 25 mg per week. This should be continued for 4 to 6 months after clinical remission to reduce the risk of relapse. For refractory forms of EF, treatment options may include biologic therapies such as tocilizumab, sirolimus (an mTOR inhibitor), or rilizumab.^[22,23]^ There is also growing interest in targeting the JAK/STAT signaling pathway and regulators of necroptosis.^[24]^ In advanced disease accompanied by joint contractures or structural deformities, surgical evaluation may be appropriate once active inflammation is suppressed. On follow-up, the patient reported notable improvements in quality of life, with diminished skin tightness and joint stiffness, and recovered function in upper limb movement and squatting. The patient has returned to performing household chores and regained complete independence in daily activities, which reinforces the initial diagnosis and supports the effectiveness of the treatment approach.

EF is an uncommon connective tissue disorder associated with substantial diagnostic difficulty. Epidemiological studies suggest that misdiagnosis or underdiagnosis occurs in 40% to 60% of cases, frequently resulting from nonspecific clinical manifestations and insufficient clinician familiarity with the disease. However, through systematic application of established international criteria (focusing on the characteristic triad of skin induration, peripheral eosinophilia, and typical imaging features) a confident diagnosis can often be reached without histopathological confirmation. Implementing a multidisciplinary team approach to synthesize these elements further improves diagnostic precision. Such a strategy not only promotes early intervention but also aids in formulating personalized management plans, ultimately enhancing patient prognosis.

## Author contributions

**Conceptualization:** Yuanhao Li, Keyan Qian.

**Project administration:** Limin Niu.

**Supervision:** Dandan Wang, Haijun Ma.

**Validation:** Minghao Guo.

**Writing – original draft:** Yongping Dang.

**Writing – review & editing:** Jin Li.
